# Ayurvedic Medicine for the Treatment of Dementia: Mechanistic Aspects

**DOI:** 10.1155/2018/2481076

**Published:** 2018-05-15

**Authors:** Akhlaq A. Farooqui, Tahira Farooqui, Anil Madan, Jolin Hwee-Jing Ong, Wei-Yi Ong

**Affiliations:** ^1^Department of Molecular and Cellular Biochemistry, The Ohio State University, Columbus, OH 43221, USA; ^2^Department of Pathology, Rajshree Medical Research Institute Bareilly, Bareilly, India; ^3^Department of Anatomy, National University of Singapore, Singapore 119260

## Abstract

Ayurvedic medicine is a personalized system of traditional medicine native to India and the Indian subcontinent. It is based on a holistic view of treatment which promotes and supports equilibrium in different aspects of human life: the body, mind, and soul. Popular Ayurvedic medicinal plants and formulations that are used to slow down brain aging and enhance memory include Ashwagandha* (Withania somnifera)*, Turmeric* (Curcuma longa)*, Brahmi* (Bacopa monnieri)*, Shankhpushpi (*Convolvulus pluricaulis, Evolvulus alsinoides*, and other species), gotu kola* (Centella asiatica)*, and guggulu (*Commiphora mukul* and related species) and a formulation known as Brāhmī Gh*ṛ*ita, containing Brahmi, Vacā* (Acorus calamus)*, Ku*ṣṭ*ha* (Saussurea lappa)*, Shankhpushpi, and Purāṇa Gh*ṛ*ita (old clarified butter/old ghee). The rationale for the utilization of Ayurvedic medicinal plants has depended mostly on traditional usage, with little scientific data on signal transduction processes, efficacy, and safety. However, in recent years, pharmacological and toxicological studies have begun to be published and receive attention from scientists for verification of their claimed pharmacological and therapeutic effects. The purpose of this review is to outline the molecular mechanisms, signal transduction processes, and sites of action of some Ayurvedic medicinal plants. It is hoped that this description can be further explored with modern scientific methods, to reveal new therapeutic leads and jump-start more studies on the use of Ayurvedic medicine for prevention and treatment of dementia.

## 1. Introduction

**Figure 1:**
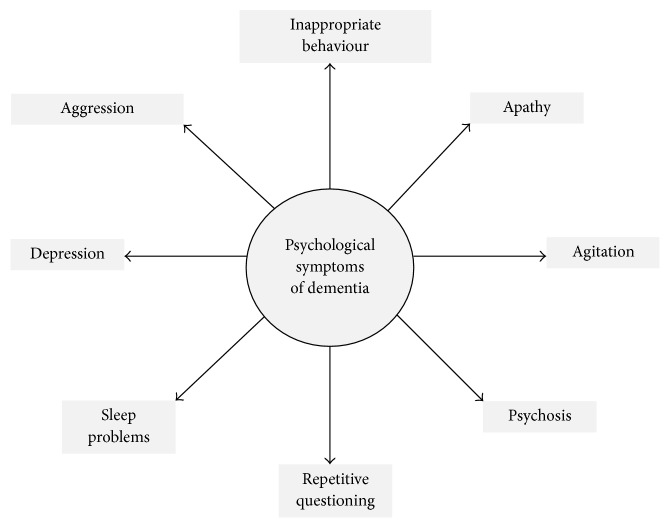
Symptoms of dementia.

Due to an increase in life expectancy, it is estimated that the number of elderly people worldwide will increase to approximately 2.1 billion by the year 2050 [1, 2]. Increase in age is a major risk factor for dementia, a clinical neurodegenerative syndrome characterized by impaired memory and activities of daily living, altered behavior, personality, and other cognitive dysfunctions [3] (Figure 1). Several types of dementia have been reported in human patients, including Alzheimer type of dementia (AD), vascular dementia, Lewy body dementia, and dementia as a result of diseases such as stroke, AIDS, and multiple sclerosis [4]. Among these, AD is the most common cause of dementia and is characterized by progressive memory loss and other cognitive deficits, including impaired judgment and decision-making, and language disturbances. In contrast, vascular dementia is defined as loss of cognitive function resulting from ischemic, hypoperfusive, or hemorrhagic brain lesions [5, 6]. Major risk factors for dementia include old age, long-term consumption of “western” diet, physical and cognitive inactivity, and epigenetic and environmental factors [7]. Other risk factors for dementia include cardiovascular and cerebrovascular problems, excessive alcohol consumption, social isolation, traumatic brain injury, and having one or two copies of the APOE*ϵ*4 genetic variant [8, 9].

Ayurvedic medicine is a personalized system of traditional medicine native to India and the Indian subcontinent. It is based on a holistic view of treatment which promotes and supports equilibrium in different aspects of human life: the body, mind, and soul [10]. Ayurveda dates back to the period of the Indus Valley civilization (about 3000 B.C.) and has been passed down through generations of oral tradition, like the other four knowledge texts (vedas) in ancient India. These include the Rigveda, Yajurveda, Samaveda, and Atharvaveda, which were composed between the 12th and 7th century B.C. Ayurvedic medicine includes descriptions of over 5000 signs and symptoms of various diseases and 700 herbs and 6,000 formulations to treat them. A direct reference to dementia in Ayurvedic literature has not been mentioned. However, the symptoms of forgetfulness and memory loss have been described [10]. Ayurvedic medicine mentions and explains the use of several herbs and their qualities for the treatment of nervous system disorders, including memory loss typically seen in older adults, but only recently have mechanistic studies been carried out, to determine the effects of these herbs on CNS disorders such as AD [11]. In recent years, there is renewed interest in the use of phytochemicals for the treatment of dementia, since pharmacological treatment of dementia using drugs (haloperidol, risperidone, aripiprazole, olanzapine, cholinesterase inhibitors, memantine, and benzodiazepines) is often inadequate and has many side effects [12–18]. The purpose of this review is to outline signal transduction processes and molecular mechanisms of some Ayurvedic medicinal plants used for the treatment of dementia. It is hoped that this description can be further explored with modern scientific validation approaches, to reveal new therapeutic leads and jump-start more studies on the use of Ayurvedic medicine for prevention and treatment of dementia.

## 2. Ayurvedic Medicinal Plants for the Treatment of Dementia

The use of complementary medicines, such as plant extracts, in dementia therapy varies according to different cultural traditions. Ayurvedic medicinal herbs modulate the neuro-endocrine-immune systems and are also rich sources of antioxidants and anti-inflammatory compounds [19, 20]. They are claimed to enhance memory and rejuvenate cognitive functions [21–23]. Several Ayurvedic medicines have been exploited for the treatment and management of acute and chronic neurological diseases. Examples of popular Ayurvedic medications include Brāhmī Gh*ṛ*ita, Divya Medha Kwath, and Brento Forte. These formulations induce specific effects on brain functions, such as increase in blood flow and maintenance of memory [11].

### 2.1. Ashwagandha

**Figure 2:**
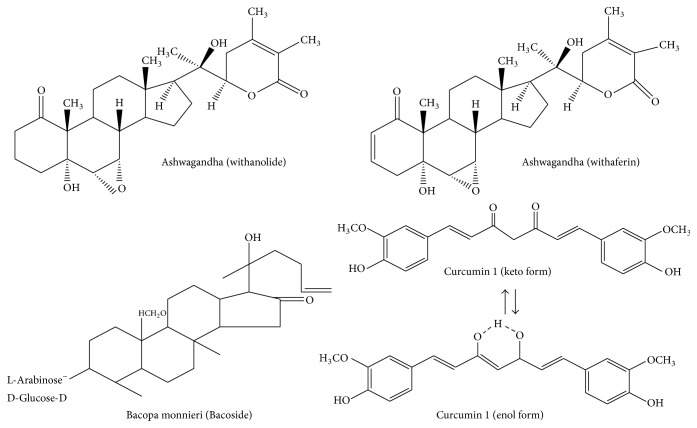
Chemical structures of withanolide, withaferin, bacoside, and curcumin.

**Figure 3:**
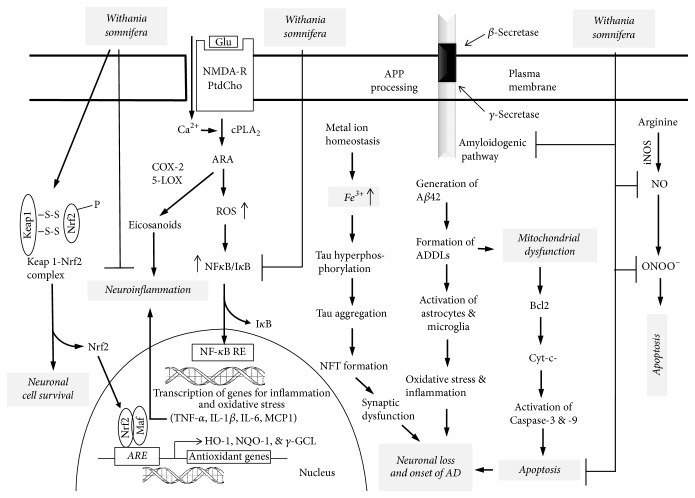
Hypothetical diagram showing target sites for the action of Ashwagandha (*Withania somnifera*). Glutamate (Glu); NMDA receptor (NMDA-R); phosphatidylcholine (PtdCho); cytosolic phospholipase A_2_ (cPLA_2_); cyclooxygenase-2 (COX-2); 5-lipoxygenase (5-LOX); arachidonic acid (ARA); reactive oxygen species (ROS); nuclear factor-*κ*B (NF-*κ*B); nuclear factor-*κ*B-response element (NF-*κ*B-RE); inhibitory subunit of NF-*κ*B (I-*κ*B); tumor necrosis factor-*α* (TNF-*α*); interleukin-1*β* (IL-1*β*); interleukin-6 (IL-6); monocyte chemoattractant protein-1 (MCP-1); nuclear factor E2-related factor 2 (Nrf2); kelch-like ECH-associated protein 1 (Keap1); antioxidant response element (ARE); small leucine zipper proteins (Maf); heme oxygenase (HO-1); NADPH quinine oxidoreductase (NQO-1); *γ*-glutamate cystein ligase (*γ*-GCL); B-cell lymphoma 2 (Bcl-2); cytochrome (cyto-c); amyloid precursor protein (APP); *β*-amyloid (A*β*); A*β*-derived diffusible ligand (ADDL); and Alzheimer disease (AD).

Ashwagandha (*Withania somnifera*, fam. Solanaceae), or Indian Ginseng, is a common herb used in Ayurvedic medicine as an adaptogen or antistress agent. Ashwagandha root contains a large variety of compounds including 12 alkaloids, 40 withanolides, and several sitoindosides and flavonoids [24–26]. Withaferin A (WL-A) and withanolide A are two constituents which show similar pharmacokinetic profiles, except that the oral bioavailability for WL-A is 1.44 times greater than that of withanolide A [27] (Figure 2). These components produce antistress, antioxidant, and immunomodulatory effects in acute models of experimental stress [28–30]. According to Ayurvedic medicine, Ashwagandha constituents provide a number of healthful effects such as youthful state of physical and mental health and increase in happiness. It is not only given to children as tonics but is consumed by the middle-aged and elderly to increase longevity [31, 32]. Recent studies have indicated that Ashwagandha root improves the body's defense against chronic diseases not only by improving cell-mediated immunity, but also through producing potent antioxidant and anti-inflammatory effects that protect against cellular damage caused by free radicals and inflammatory mediators [31, 32]. At the molecular level, Ashwagandha root may produce beneficial effects in AD by inhibiting the activation of NF-*κ*B, blocking *β*-amyloid (A*β*) production, reducing apoptotic cell death, restoring synaptic function, and enhancing antioxidant effects through the migration of Nrf2 to the nucleus, where it increases the expression of antioxidant enzymes [33] (Figure 3). It is suggested that WL-A activates the translocation of Nrf2 to the nucleus, where the transcription factor upregulates the expression of neuroprotective proteins, such as heme oxygenase-1 [34, 35]. Treatment of human neuroblastoma SK-N-SH cells with methanolic extracts of Ashwagandha root results in dendrite extension, neurite outgrowth, and synapse formation [36, 37]. Moreover, treatment of cultured rat cortical neurons with A*β* (25–35) (10 *μ*M) produces axonal and dendritic atrophy and pre- and postsynaptic loss, and these changes were abrogated by treatment with WL-A (1 *μ*M) [37]. WL-A also attenuates the expression of semaphorin 3A to facilitate neural regeneration. The beneficial effects of Ashwagandha root constituents in neurodegenerative diseases may be due to their neurite promoting, antioxidant, anti-inflammatory, antiapoptotic, and anxiolytic activities, as well as their ability to improve mitochondrial dysfunction and restore energy levels and increase levels of antioxidant defenses such as reduced glutathione [38] (Figure 3).

In animals, WL-A (10 *μ*mol kg^−1^ day^−1^, for 13 days, p.o.) restores A*β* (25–35)-induced memory deficit in mice and recovers the decline of axons, dendrites, and synapses in the cerebral cortex and hippocampus. Based on the above information, it is proposed that WL-A is an important candidate for the treatment of dementia and neurodegenerative diseases, since it is able to repair damaged neuronal networks [33, 38]. Ashwagandha is a safe herb [39, 40], although a few people experience diarrhea or nausea after consuming the root. It should not be taken with barbiturate-type sedatives, since the herb can increase the effectiveness of these drugs. Ashwagandha can cross the blood-brain barrier and lower inflammation in the brain.

The half-lives of Ashwagandha in the circulation and the brain are not known. Large multicenter clinical trials of Ashwagandha in patients with dementia have not been performed. A preliminary study indicates that* Withania somnifera* (500 mg/day) added adjunctively to medications improves auditory-verbal working memory, reaction time, and social cognition in patients with bipolar disorders [41].

### 2.2. Turmeric

**Figure 4:**
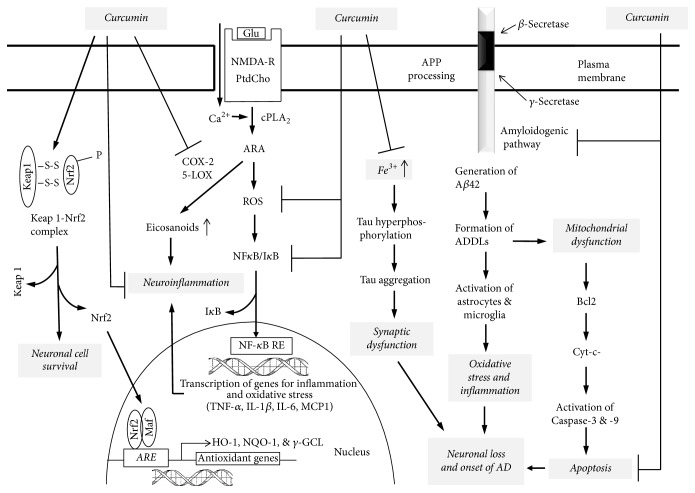
Hypothetical diagram showing target sites for the action of curcumin (diferuloylmethane) in signal transduction pathway. Glutamate (Glu); NMDA receptor (NMDA-R); phosphatidylcholine (PtdCho); cytosolic phospholipase A_2_ (cPLA_2_); cyclooxygenase-2 (COX-2); 5-lipoxygenase (5-LOX); arachidonic acid (ARA); reactive oxygen species (ROS); nuclear factor-*κ*B (NF-*κ*B); nuclear factor-*κ*B-response element (NF-*κ*B-RE); inhibitory subunit of NF-*κ*B (I-*κ*B); tumor necrosis factor-*α* (TNF-*α*); interleukin-1*β* (IL-1*β*); interleukin-6 (IL-6); monocyte chemoattractant protein-1 (MCP-1); nuclear factor E2-related factor 2 (Nrf2); kelch-like ECH-associated protein 1 (Keap1); antioxidant response element (ARE); small leucine zipper proteins (Maf); heme oxygenase (HO-1); NADPH quinine oxidoreductase (NQO-1); *γ*-glutamate cystein ligase (*γ*-GCL); B-cell lymphoma 2 (Bcl-2); cytochrome (cyto-c); amyloid precursor protein (APP); *β*-amyloid (A*β*); A*β*-derived diffusible ligand (ADDL); and Alzheimer disease (AD).

Curcumin (C_21_H_20_O_6_) or diferuloylmethane (bis-*α*,*β*-unsaturated *β*-diketone) (Figure 2) is a hydrophobic polyphenolic compound (mol mass of 368.38) present in turmeric (an ingredient in curry powder). It is derived from the rhizome of* Curcuma longa*, which belongs to the family Zingiberaceae. It has antioxidant, anti-inflammatory, and cancer chemopreventive properties [42]. Curcumin reduces oxidative damage and improves cognitive functions related to the aging process. It induces antioxidant effects by modulating the Nrf2-keap1 pathway and reduces genomic instability events [43]. Nrf2 is primarily present in the cytoplasm, where it is bound with the Kelch-like ECH-associated protein 1 (Keap1). Interaction of curcumin with Keap 1 releases Nrf2, which migrates into the nucleus and binds as a heterodimer to antioxidant responsive elements in DNA, to initiate target gene expression. Nrf2-regulated genes include antioxidant enzymes, molecular chaperones, DNA repair enzymes, and anti-inflammatory response proteins [44] (Figure 4). These proteins promote the reduction in ROS generation while increasing the ability of the cell to repair any subsequent damage [44, 45]. Curcumin also suppresses proinflammatory pathways by blocking the production of TNF-*α*, IL-1*β*, and other proinflammatory cytokines, including IL-8, MIP-1*β*, and MCP-1, in astrocytes and microglia. Curcumin attenuates neuroinflammation through the inhibition of phospholipase A_2_ (PLA_2_) and cyclooxygenase (COX-2) enzymes associated with the metabolism of neural membrane phospholipids to prostaglandins (Figure 4). It reduces glial fibrillary acidic protein (GFAP) expression, improves spatial memory in the A*β*-induced rat model of AD, and decreases GFAP and COX-2 expression in A*β*-treated astrocytes [46]. Both* in vitro* and* in vivo* studies indicate that curcumin binds with A*β* and inhibits its aggregation [43, 47], as well as fibril and oligomer formation [43].* In vivo* studies have shown that dietary curcumin not only crosses the blood-brain barrier and decreases A*β* deposition in AD transgenic mice [43], but also markedly inhibits Tau phosphorylation [48]. The absorption rate and bioavailability of curcumin can be increased by consuming it with black pepper* (Piper nigrum)*. Studies have indicated that piperine, an active ingredient in black pepper, increases the bioavailability and bioefficacy of curcumin by inhibiting its glucuronidation [49]. Interestingly, consumption of piperine and curcumin has been found to protect against chronic unpredictable stress-induced cognitive impairment and oxidative damage in mice [50, 51].

The half-lives of curcumin in the circulation and the brain are not known. Large multicenter clinical trials of curcumin in patients with dementia have not been performed, although a number of small studies have been conducted in healthy individuals. Curcumin (400 mg/day) significantly improves performance on sustained attention and working memory tasks, compared with placebo, in healthy adults over 60 years of age [52]. Another study indicates that treatment with curcumin (1500 mg/day) results in no loss of cognition in the treatment group, whereas loss of cognition is found in the placebo group, among community-dwelling older adults [53].

### 2.3. Brahmi* (Bacopa monnieri)*

**Figure 5:**
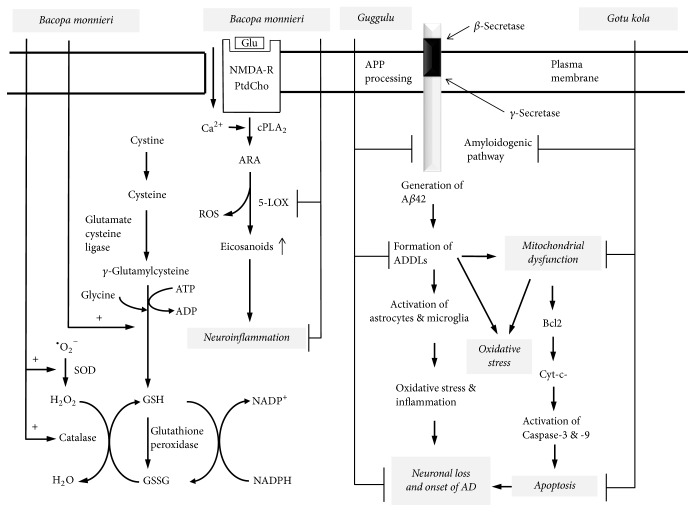
Hypothetical diagram showing target sites for the action of Bacopa monnieri, guggulu, and gotu kola. Plasma membrane (PM); *β*-amyloid (A*β*); A*β*-derived diffusible ligand (ADDL); B-cell lymphoma 2 (Bcl-2); cytochrome (cyto-c); amyloid precursor protein (APP); Alzheimer disease (AD); reduced glutathione (GSH); oxidized glutathione (GSSG).

*Bacopa monnieri* belongs to the family Scrophulariaceae and is found throughout the Indian subcontinent in wet, damp, and marshy areas [54]. It has many branches with small oblong leaves and purple flowers. This plant is not only used for the treatment of a number of nervous system disorders such as insomnia, anxiety, and epilepsy, but also used for enhancing memory and the intellect [55]. In Ayurvedic medicine,* Bacopa monnieri* is used as a memory enhancing, anti-inflammatory, analgesic, antipyretic, sedative, and antiepileptic agent, which acts as a nootropic (repairing damaged neurons and improving brain function). According to Ayurvedic medical practitioners, the memory enhancing properties of* Bacopa monnieri* are due to the presence of bacoside A, assigned as 3-(a-l-arabinopyranosyl)-O-*β*-d-glucopyranoside-10, 20-dihydroxy-16-keto-dammar-24-ene [56], and bacoside B [57] (Figure 5).

Bacosides inhibit lipoxygenase activity and scavenge free radicals. They protect neural cells of the prefrontal cortex, hippocampus, and striatum against cytotoxicity and DNA damage implicated in AD. Bacosides increase glutathione peroxidase, chelate iron [58, 59], and enhance nitric oxide-mediated cerebral vasodilation, leading to improvements in total memory score [59]. Bacosides may also act by regulating membrane phosphorylation/dephosphorylation processes [60]. This leads to an increase in protein and RNA turnover in certain brain regions such as the hippocampus [57]. Furthermore, the combination of bacosides A and B not only induces antistress effects [61], but also protects the brain against smoking-induced membrane damage [62] and d-galactosamine-induced liver injury [63]. While there are no studies that prove that* Bacopa monnieri* causes side effects, it has been observed that excessive intake of* Bacopa monnieri* may lead to stomach upset, diarrhea, and nausea. The intake and use of* Bacopa monnieri* should be avoided by pregnant and breastfeeding women. To reduce the risk of side effects, it would be a good idea to gauge an individual's tolerance for this herb.

The half-lives of bacosides in the circulation and the brain are not known. A number of clinical trials have been carried out in human subjects on the use of* Bacopa monnieri *for improving cognition.* Bacopa monnieri* (2 × 150 mg) treatment for 90 days improves performance in a spatial working memory task in healthy humans [64, 65].* Bacopa monnieri* treatment for 3 months decreases the rate of forgetting of newly acquired information in human subjects between 40 and 65 years of age [55]. Standardized* Bacopa monnieri* extract treatment (300 mg/day) for 12 weeks improves performance in a delayed recall and Stroop Task (assessing the ability to ignore irrelevant information), in participants without dementia aged 65 and above [66]. Moreover, treatment with* Bacopa monnieri* (300 mg/day) improves verbal learning, memory acquisition, and delayed recall in healthy volunteers over 55 years of age [67].

*Bacopa monnieri* is used in a formulation known as Brāhmī Gh*ṛ*ita, containing Brahmi* (Bacopa monnieri),* Vacā* (Acorus calamus)*, Ku*ṣṭ*ha* (Saussurea lappa)*, Shankhpushpi* (Convolvulus pluricaulis)*, and Purāṇa Gh*ṛ*ita (old clarified butter/old ghee). The roots and rhizomes of* Acorus calamus* are used in Ayurvedic medicine on a regular basis for the treatment of insomnia, melancholia, neurosis, loss of memory, and remittent fevers.* Convolvulus pluricaulis* has been shown to improve learning and memory in rodents [68].* Saussurea lappa* has been reported to produce anti-inflammatory activity [69]. Old clarified butter or old ghee is described in Ayurveda as a memory enhancer, anticonvulsant, and anti-inflammatory agent [70, 71]. This formulation is used for the treatment of a number of neurological disorders such as anxiety and dementia [72]. The molecular mechanisms associated with the beneficial effects of Brāhmī Gh*ṛ*ita are not fully understood. However, it is reported that Brāhmī Gh*ṛ*ita may act not only by reversing cholinergic deficits in the frontal cortex and hippocampus [73], but also via alleviating cholinergic neurodegeneration [74], lowering norepinephrine, and increasing 5-hydroxytryptamine levels in the hippocampus, hypothalamus, and cerebral cortex [75]. The rhizome of* Acorus calamus*, another constituent of Brāhmī Gh*ṛ*ita, is used as a brain tonic for improving memory and treatment of epilepsy. Methanolic extracts of* Acorus calamus *roots contain the essential oil *β*-asarone, which inhibits acetylcholinesterase [76]. Finally, old clarified butter is especially good for healing the mind [77]. The half-lives of Brāhmī Gh*ṛ*ita in the circulation and the brain are not known. Large multicenter clinical trials of Brāhmī Gh*ṛ*ita in patients with dementia have not been performed.

### 2.4. Shankhpushpi

Shankhpushpi* (Convolvulus pluricaulis)* is a common plant in India. It belongs to the family Convolvulaceae. The whole plant of Shankhpushpi is used in various formulae as a nervine tonic for improvement of memory and cognitive function [78, 79]. Shankhpushpi is recommended for nervous system disorders, such as stress, anxiety, mental fatigue, and insomnia [79–81]. It has been suggested that Shankhpushpi has a calming effect by regulating the body's production of the stress hormones, adrenaline and cortisol [82]. The major bioactive components of* Convolvulus pluricaulis* are glycosides, flavonoids, coumarins, anthocyanins, and alkaloids. Sitosterol glycoside, octacosanol tetracosane, hydroxycinnamic acid, and glucose have also been isolated from the plant [83]. These metabolites contribute to its nootropic and memory enhancing properties, along with its other pharmacological activities [79, 82, 84]. Ethanolic extracts of Shankhpushpi improve learning and memory and induce antioxidant effects in rats [68]. Furthermore, ethanolic extracts of the whole Shankhpushpi plant, when administered to cholesterol-fed gerbils, induce reduction in serum levels of cholesterol, LDL cholesterol, triglycerides, and phospholipids [79]. The administration of ethanolic extracts of Shankhpushpi increases acetylcholine content in fields CA1 and CA3 of the hippocampus in a dose-dependent manner [85, 86]. This is accompanied by a significant increase in the number of dendritic intersections, branch points, and dendritic processes arising from the cell bodies of neurons, in comparison with age-matched saline controls. Results suggest that ethanolic extracts of Shankhpushpi enhance memory by increasing neurite outgrowth [86, 87].

### 2.5. Gotu kola

Gotu kola* (Centella asiatica)* is another herb that is known as Brahmi (besides* Bacopa monnieri*). It belongs to the family Apiaceae and is a perennial creeping herb with long thick stems and smooth fan leaves. It is widely used as a blood purifier and for treating high blood pressure, enhancing memory, and promoting longevity. Tea made from gotu kola can be very helpful for relieving tension, relaxing the mind, and soothing anxiety. As a nervine adaptogen, constituents of gotu kola are capable of increasing intelligence, longevity, and memory. In the Ayurvedic system of medicine, water extracts of gotu kola are used not only for rejuvenating and restoring neural cells, but also for stimulating healthy sleep. It has a powerful effect on quality of life in disorders such as epilepsy. The primary active ingredients of gotu kola are saponins (also called triterpenoids), which include asiaticosides, in which a trisaccharide moiety is linked to the aglycone asiatic acid, madecassoside, and madasiatic acid [88]. Other components isolated from* Centella asiatica* such as brahmoside and brahminoside may be responsible for CNS and uterorelaxant actions but have yet to be confirmed by clinical studies. At the molecular level, asiaticoside derivatives from gotu kola (asiatic acid and asiaticoside) are capable of reducing hydrogen peroxide-induced cell death, decreasing free radical levels, and inhibiting A*β*-mediated neural cell death* in vitro*. Results suggest a role for gotu kola in the prevention and treatment of A*β* toxicity and AD type of dementia (Figure 5) [89–91]. Gotu kola extracts possess antioxidant activity and can alter mitochondrial function [92, 93]. Because mitochondrial dysfunction is a common process that contributes to neurodegeneration in many neurodegenerative diseases [94], there are potentially broad implications for the use of water extracts of gotu kola.

In animals, water extracts of gotu kola attenuate cognitive impairment in the Tg2576 mouse model of A*β* accumulation without altering plaque burden [95] and can prevent A*β* toxicity* in vitro* [90]. Gotu kola has a safe record [96]. However, at high doses, it makes consumers drowsy. The half-lives of gotu kola in the circulation and the brain are not known. Large multicenter clinical trials of gotu kola in patients with dementia have not been performed. Studies on a few healthy human adults have shown promising cognitive-enhancing effects of gotu kola extracts [97, 98]. One study reports that treatment with* Centella asiatica* (750 mg/day) for 2 months enhances working memory and improvements in self-rated mood in healthy elderly volunteers [98].

### 2.6. Guggulu

Guggulu is an oleogum resin exuding from cracks and fissures or incisions in the bark of several plant species including* Commiphora mukul, Commiphora molmol, Commiphora abyssinica, Commiphora Burseraceae*, and* Commiphora wightii*. It is pale yellow or brown in color with an aromatic odor and bitter astringent taste [99]. Guggulu preparations contain 30% to 60% water-soluble gum, 20% to 40% alcohol-soluble resins, and about 8% volatile oils, which have many biological activities. Water-soluble extracts of guggulu contain mucilage, sugars, and proteins. Alcohol-soluble extracts of guggulu contain commiphoric acids, commiphorinic acid, and heerabomyrrhols. The volatile constituents of guggulu include terpenes, sesquiterpenoids, cuminic aldehyde, eugenol, the ketone steroids Z- and E-guggulsterone, and guggulsterols I, II, and III [100]. Guggulu contains ferulic acids, phenols, and other nonphenolic aromatic acids which are potent scavengers of superoxide radicals and can be important for the treatment of neurodegenerative diseases that are associated with oxidative stress [101, 102]. In addition, guggulsterones antagonize nuclear hormone receptors and decrease cholesterol levels, which may explain the hypolipidemic effects of guggulu extracts [103]. Many studies have indicated that there is a link between cholesterol, amyloid precursor protein processing, and AD [104, 105]. Cholesterol is an essential modulator of the physicochemical state and functional activity of the cell membrane and thus plays an essential role in the regulation of synaptic function and neuronal plasticity.* In vitro* and* in vivo* modulation of membrane cholesterol levels affect different cholesterol pools within the plasma membrane bilayer that are differentially sensitive to the disrupting effect of A*β* [106]. It is likely that beneficial effects of guggulu on AD may be due to its cholesterol-lowering effects [104]. Decreased neuronal cholesterol levels, in turn, inhibit the A*β*-forming amyloidogenic pathway, possibly by removing amyloid precursor protein from cholesterol and sphingolipid-enriched membrane microdomains. These intriguing relationships raise hopes that cholesterol-lowering strategies may influence the progression of dementia associated with AD [104, 106]. Administration of guggulipid (Z-guggulsterone) significantly lowers both serum LDL cholesterol and triglyceride levels, supporting the view that guggulipids may produce beneficial effects in the cardiovascular system [107].

In animals, Z-guggulsterone attenuates behavioral abnormalities induced by neuroinflammation in the forced swim and tail suspension tests [108] and prevents memory impairment in the scopolamine-induced memory impairment model, through activation of the CREB-BDNF signal [109]. Guggulipids also produce beneficial effects in the streptozotocin-induced memory deficit model of dementia, which can be attributed to their cholesterol-lowering, antioxidant, and antiacetylcholine esterase activities [107]. The half-lives of guggulu compounds in the circulation and the brain are not known. Large multicenter clinical trials of guggulu compounds in patients with dementia have not been performed.

## 3. Conclusion

Popular Ayurvedic medicinal plants (Ashwagandha, Turmeric, Brahmi, Shankhpushpi, gotu kola, and guggulu) not only reduce brain aging and induce antistress and memory enhancing effects which help in regeneration of neural tissues, but also induce antioxidant, anti-inflammatory, antiamyloidogenic, nutritional, and immune-supportive effects in the human body. Scientific validation and the documentation of Ayurvedic medicines are essential for their quality evaluation and global acceptance. Therapeutic efficacy of Ayurvedic herbal formulations might be enhanced, not only by achieving purity, but also through a better understanding of their biological effects. These days attempts are underway to achieve this goal. Once it is done, large multicenter clinical trials of Ayurvedic medicine can be planned and performed in patients with dementia and other neurodegenerative disorders.
